# Waardenburg Syndrome: The Contribution of Next-Generation Sequencing to the Identification of Novel Causative Variants

**DOI:** 10.3390/audiolres14010002

**Published:** 2023-12-21

**Authors:** William Bertani-Torres, Karina Lezirovitz, Danillo Alencar-Coutinho, Eliete Pardono, Silvia Souza da Costa, Larissa do Nascimento Antunes, Judite de Oliveira, Paulo Alberto Otto, Véronique Pingault, Regina Célia Mingroni-Netto

**Affiliations:** 1 Centro de Estudos sobre o Genoma Humano e Células Tronco, Departamento de Genética e Biologia Evolutiva, Instituto de Biociências, Universidade de São Paulo, São Paulo 05508-090, Brazil; wiilliiam@hotmail.com (W.B.-T.); silvia.costa@ib.usp.br (S.S.d.C.); larissannascimento@gmail.com (L.d.N.A.); otto@usp.br (P.A.O.); 2 Department of Embryology and Genetics of Malformations, INSERM (Institut National de la Santé et de la Recherche Médicale) UMR (Unité Mixte de Recherche) 1163, Université Paris-Cité and Institut Imagine, 75015 Paris, France; veronique.pingault@inserm.fr; 3 Otorhinolaryngology Lab-LIM 32, Hospital das Clínicas, Faculdade de Medicina, Universidade de São Paulo, São Paulo 01246-000, Brazil; lezi.karina@gmail.com (K.L.); danillo.coutinho92@gmail.com (D.A.-C.); 4 Instituto de Ciências da Saúde, Universidade Paulista UNIP, São Paulo 04026-002, Brazil; e_pardono@yahoo.com.br; 5 Colégio Miguel de Cervantes, São Paulo 05618-001, Brazil; 6 Médecine Génomique des Maladies Rares, AP-HP, Hôpital Necker-Enfants Malades, 75015 Paris, France; judite.de-oliveira@aphp.fr

**Keywords:** Waardenburg syndrome, NGS, neurocristopathy

## Abstract

Waardenburg syndrome (WS) is characterized by hearing loss and pigmentary abnormalities of the eyes, hair, and skin. The condition is genetically heterogeneous, and is classified into four clinical types differentiated by the presence of dystopia canthorum in type 1 and its absence in type 2. Additionally, limb musculoskeletal abnormalities and Hirschsprung disease differentiate types 3 and 4, respectively. Genes *PAX3*, *MITF*, *SOX10*, *KITLG*, *EDNRB*, and *EDN3* are already known to be associated with WS. In WS, a certain degree of molecularly undetected patients remains, especially in type 2. This study aims to pinpoint causative variants using different NGS approaches in a cohort of 26 Brazilian probands with possible/probable diagnosis of WS1 (8) or WS2 (18). DNA from the patients was first analyzed by exome sequencing. Seven of these families were submitted to trio analysis. For inconclusive cases, we applied a targeted NGS panel targeting WS/neurocristopathies genes. Causative variants were detected in 20 of the 26 probands analyzed, these being five in *PAX3*, eight in *MITF*, two in *SOX10*, four in *EDNRB*, and one in *ACTG1* (type 2 Baraitser-Winter syndrome, BWS2). In conclusion, in our cohort of patients, the detection rate of the causative variant was 77%, confirming the superior detection power of NGS in genetically heterogeneous diseases.

## 1. Introduction

Waardenburg syndrome (WS) is a genetic condition characterized by an association of sensorineural hearing impairment/loss and pigmentary abnormalities of the hair, eyes, and skin. The pigmentary abnormalities are usually congenital but some may result from early depigmentation [1]. The overall population frequency of WS is estimated to be around 1/42,000 [2]. The syndrome is divided into four clinical types, all resulting from embryonic neural crest cell (NCC) defects in the differentiation, proliferation, survival, and migration of their derivatives [3]. Type 1 (WS1, MIM# 193500) presents the most remarkable feature of WS, which is dystopia canthorum (DC), an increase in the inner canthi distance of the eyes. Type 2 (WS2, MIM# 193510) is differentiated from type 1 by the absence of DC, presenting therefore only deafness associated with pigmentary abnormalities. Further diagnostic criteria for distinguishing types I and II have been proposed by the Waardenburg consortium [4,5] and by Pardono et al. (2003) [6]. Type 3 (WS3, MIM# 148820), also known as Klein–Waardenburg, is rarer and more severe, having the same facial dysmorphic features as type 1 with the addition of musculoskeletal abnormalities of upper limbs. Type 4 (WS4, MIM# 277580), also known as Shah–Waardenburg, is characterized by the association of deafness and pigmentary abnormalities with Hirschsprung disease (HD) and/or other intestinal/neural abnormalities [7].

Six genes are known to be involved in the causation of WS: *PAX3* (paired box 3), *MITF* (melanocyte inducing transcription factor), *SOX10* (SRY-Box transcription factor 10), *EDN3* (endothelin 3), *EDNRB* (endothelin receptor type B) and *KITLG* (KIT ligand). It is well established that *PAX3* is involved with types 1 and 3, most cases being caused by pathogenic variants in heterozygosis [8,9,10], while type 3 has been reported as a consequence of pathogenic variants in the homozygous state, with more severe manifestations [11,12]. WS2 is typically caused by pathogenic heterozygous variants in *MITF* and *SOX10*, and, to a lesser degree, in *EDNRB* [10,13], and also in heterozygous [14] and homozygous states in *KITLG* [15]. In cases of WS4, approximately half are caused by heterozygous *SOX10* pathogenic variants [16,17,18]. Another 20 to 30 percent of the cases are explained by pathogenic variants in genes of the endothelin pathway, *EDNRB*, and *EDN3*, both with dominant or recessive modes of inheritance (in some instances, cases of incomplete penetrance, as reported by [13,16,19]). A neurological variant of type 4, named PCWH (peripheral demyelinating neuropathy, central dysmyelination, Waardenburg syndrome with Hirschsprung disease) is caused exclusively by pathogenic variants in *SOX10* with a predominance of truncating variants on the last exon of the gene [20,21]. Other details about WS gene functions are presented and discussed by Pingault et al. (2010) [10], Zazo Seco et al. (2015) [14], and Issa et al. (2017) [13].

Since 2017, with the association of mono-allelic variants of *EDNRB* in WS2, no new gene related to the molecularly unsolved parcel of clinically diagnosed WS patients, especially those of type 2 WS, has been reported. The last important new gene discovery associated with WS took place in 2015 with the finding of one *KITLG* variant (confirmed in 2022) and three other variants in four different families, in a limited number of affected patients [14,15].

Molecular testing for genetic heterogeneous diseases caused by variants in different genes, especially when structural variants are frequent and when there might be unidentified causative genes, turns next-generation sequencing (NGS) into a very advantageous option for a high rate of molecular diagnosis. Even though WS genes contain few exons, copy number variations (CNV) and other structural variants are not detected by Sanger sequencing [16,22,23,24,25,26,27,28,29,30], therefore requiring further application of other tests such as multiplex ligation-dependent probe amplification (MLPA) or array-CGH, thus increasing costs. NGS, though usually also expensive, is a more suitable and affordable option for these cases. The technique analyzes many genes; it is also capable, although it may not be the best technique, of detecting CNVs in one single run [31,32].

In this study, DNA samples from 26 index cases with incomplete previous analysis of WS genes were submitted to exome sequencing followed, in a subset of samples, by an NGS panel to allow better coverage of difficult regions. Variants were sorted out with standard filtering strategies to locate rare pathogenic candidate variants. By applying this strategy, we were able to find the molecular explanation for 20 index cases (77% of our sample). Among these cases, a patient formerly misdiagnosed as WS1, carries a de novo variant in *ACTG1*, associated with type 2 Baraitser-Winter syndrome (BWS2).

## 2. Materials and Methods

The molecular cause of WS was investigated in samples from 26 patients clinically diagnosed with WS, these being eight probands with WS1 (3 familial and 5 isolated sporadic cases) and 18 probands with WS2 (9 familial and 9 isolated sporadic). All patients presented signs of the proposed diagnostic criteria of the Waardenburg consortium [4,5] and were evaluated by at least one medical geneticist. Our cohort partially comprises unsolved cases described in [6,22,23]. It also includes new cases ascertained in Centro de Estudos sobre o Genoma Humano e Células Tronco, São Paulo, Brazil. Blood samples (5 mL) or buccal swabs were collected for DNA extraction following informed consent under a protocol approved by the Instituto de Ciências Biomédicas, University of São Paulo (Protocol CEP 288/1998). Patients seen more recently at our institute equally agreed to participate by signing an informed consent form approved by the Ethics Committee in Research—Human Beings (APPROVAL 1.133.416—23 June 2015) of the Instituto de Biociências, University of São Paulo and also Protocol CAAE: 47637821.0.0000.5464 approved by CONEP (the National Council of Ethics in Research), in 2022. All protocols are in accordance with the Declaration of Helsinki.

Not all of the five known WS genes (*PAX3*, *MITF*, *SOX10*, *EDNRB*, *EDN3*) had been previously investigated by Sanger sequencing in all samples, with partial analysis in many of them. Most WS1 patient samples had at least the full *PAX3* gene investigated by Sanger sequencing. For primer description and methodology used, see Bocangel et al. (2018) [23] and Batissoco et al. (2022) [22]. 

MLPA was performed in 20 cases, four cases of WS1 (2 familial and 2 isolated) and in sixteen of the WS2 cases (7 familial and 9 isolated), with probes for *PAX3*, *MITF*, and *SOX10* (*KIT* SALSA P186-B1 MRC Holland, Amsterdam, The Netherlands).

In this work, the platform for Array-CGH CytosureTM, ISCA v2 array 4X180K” (OGT, Oxfordshire, UK) of 180,000 oligonucleotides with a resolution of approximately 50–100 Kb. was used in two of the isolated WS1 patients and nine (3 isolated and 6 familial) of the WS2 patients.

For whole exome sequencing (WES), one μg of DNA of the samples was used. In seven cases, samples of the mother and the father were also sent for WES to allow trio analysis. DNA was fragmented enzymatically and the library was prepared and enriched by *KIT* SureSelectQXT Target Enrichment (Agilent Technologies, Santa Clara, CA, USA). The capture and analysis of the amplified fragments (quantity and size) were performed both with the Bioanalyzer 2100 (Agilent Technologies, Santa Clara, CA, USA) and by real-time PCR (Thermo Fisher Scientific, Waltham, MA, USA). Finally, sequencing was performed using the “HiSeq 2500” (Illumina, San Diego, CA, USA). Reads were aligned to the reference genome (hg19) with the “Burrows-Wheeler Aligner” (BWA) [33]. 

Further manipulations and quality control were performed with Picard (Broad Institute, Cambridge, MA, USA). The VCF generation was performed using GATK (Broad Institute, Cambridge, MA, USA) and annotated with Annovar (annovar.openbioinformatics.org/en/latest/—accessed on 18 July 2019) [34]. Variants were annotated for population data with standard public databases (gnomAD, ClinVar, ExAC, 1000 genomes, NHLBI Exome Sequencing Project (ESP)) and also a Brazilian database ABraOM [35]. The evaluation of the quality of the exome was made from the FastQ files. The files were analyzed using the SureCall program (Agilent Technologies, Santa Clara, CA, USA). The mean vertical coverage of the target regions for these patients was 59.04 reads.

After all of the aforementioned techniques were applied, nine unsolved cases were selected to be investigated in a targeted NGS panel, to allow better coverage of difficult regions. This NGS panel used a custom-designed *KIT* targeting genes associated with WS and genes associated with syndromes for differential diagnosis inclunding the genes *EDN3*, *EDNRB*, *KIT*, *KITLG*, *MITF*, *PAX3*, *SNAI2*, *SOX10*. This panel covers exons, splice consensus sequences, and some regulatory regions. An amount of 50ng gDNA was sheared by using an enzymatic DNA fragmentation with the Twist Library Preparation EF *KIT* 1 according to the manufacturer’s sample preparation protocol, then hybridized to a target-specific probe using a custom-designed Agilent SureSelect XT HS2 *KIT* (Agilent Technologies, Santa Clara, CA, USA) under probe-specific hybridization conditions. Sequencing was performed on an Illumina “NextSeq500” (Illumina, San Diego, CA, USA) machine. The mean value for bases covered more than a hundred times by the panel was 98.7%. Variants were visualized and filtered using the Polyweb online platform interface, designed by the Bioinformatics platform of the Université Paris-Cité. 

Besides the preliminary analysis of some WS-related genes, Sanger sequencing was used to confirm the presence of candidate variants found through WES or the NGS panel and to study the segregation of these variants in families, when samples were available. As mentioned, primer description and methodology are described by [22,23]. Additionally, in the two cases with *PAX3* duplication and *EDNRB* deletion, additional primer pairs were designed to analyze breakpoints and segregation. The following primers were used in *PAX3* duplication: 6F—CGCCCAAACAACACAGAAGG and 6dupR—ATGTGATAGGTACGTTCAGGAC; in the case of *EDNRB* deletion three primers were used: 8F—ACTGAAAGAAAGGGCCCAAG, 8R—TTTTAATAGTGTGCTGTGCAAATAC, and 8delR—AGCTCATGCCTGAACGAAGC. The qPCR protocols are described in [22]. 

## 3. Results

**Figure 1:**
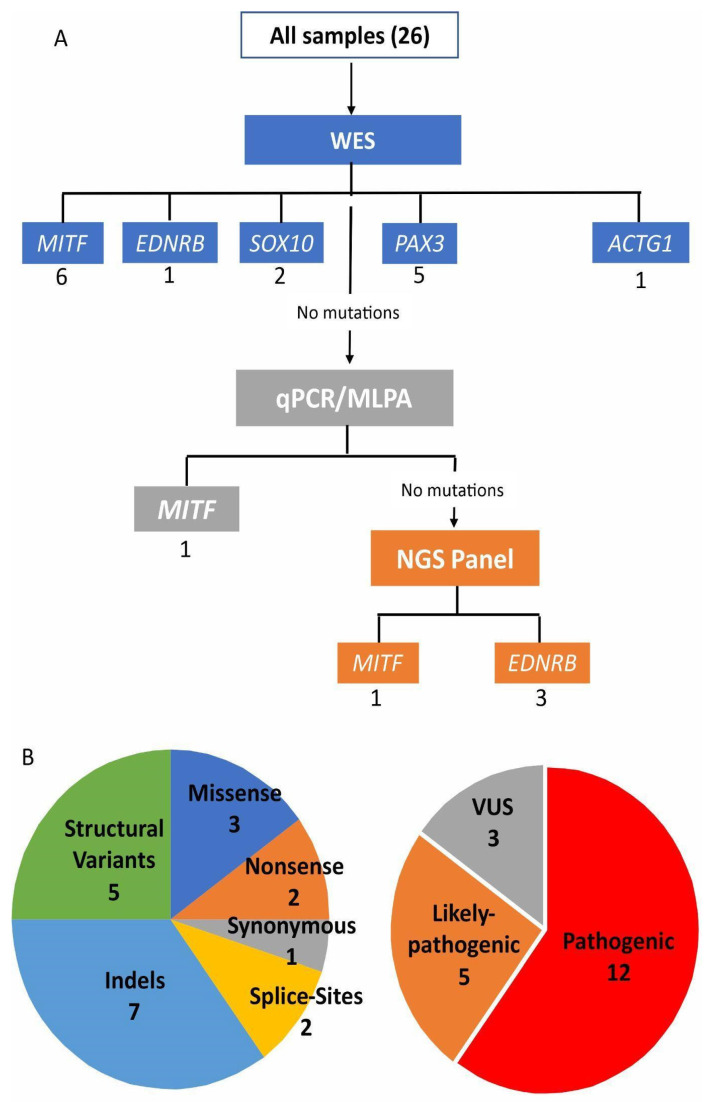
(**A**) Combination of methodologies and molecular strategy to diagnose a cohort of clinically suspected Waardenburg syndrome patients. The number of causative variants found in each gene through each methodology is written below each gene name. In (**B**) two plots represent the types of variants found.

The combination of methodologies used in this work (Figure 1A,B) was able to detect candidates to causative variants in 20 patients (13 were classified as pathogenic, five likely pathogenic, and two variants of unknown significance): 19 within the known genes of WS, and one patient was reclassified with BWS2 due to a de novo variant in *ACTG1*, resulting in a final detection rate of 77% (20/26) (Table 1). A cohort summary including phenotypes and previous or additional molecular analysis is presented in Table 2. Separately, initial WES detected the molecular cause in 15 out of the 26 patients (57%). Within this technique, trio analysis was possible for seven families (1 WS1; 1 WS1 > BSW2; 1 WS1 > WS2; 4 WS2). The trio analysis strategy indicated the molecular cause in one WS1 patient with a variant in *PAX3* and the case of the WS1 > BSW2 patient mentioned above. MLPA was used to confirm a *MITF* deletion predicted by the qPCR screening, correctly detecting the molecular cause in another patient (Batissoco et al., 2022—patient W14). In nine cases (four with previous trio analysis), a targeted NGS panel detected the molecular cause in four additional patients (44%). Among these four cases, only one had previous trio analysis (LGH11). 

Among the 20 molecularly solved cases, eight are single-nucleotide substitutions (SNVs): three missense substitutions, two nonsense variants, one synonymous variant, and two affecting splice sites. Seven cases are indels (smaller than 50 bp) (Table 1 and Figure 1B). 

**Figure 2:**
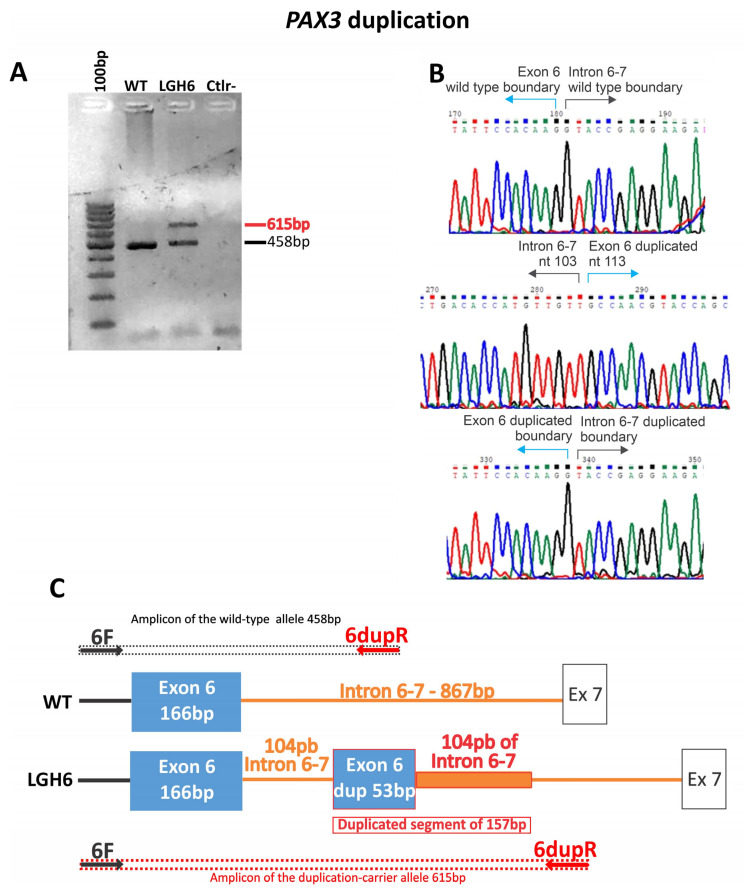
Illustration of the *PAX3* duplication in case LGH6. (**A**) Gel electrophoresis of the PCR product of the exon 6 of *PAX3*. (**B**) Sanger sequencing of the PCR product carrying the duplication. (**C**) Schematic representation of the duplication. Arrows indicate primer location.

**Figure 3:**
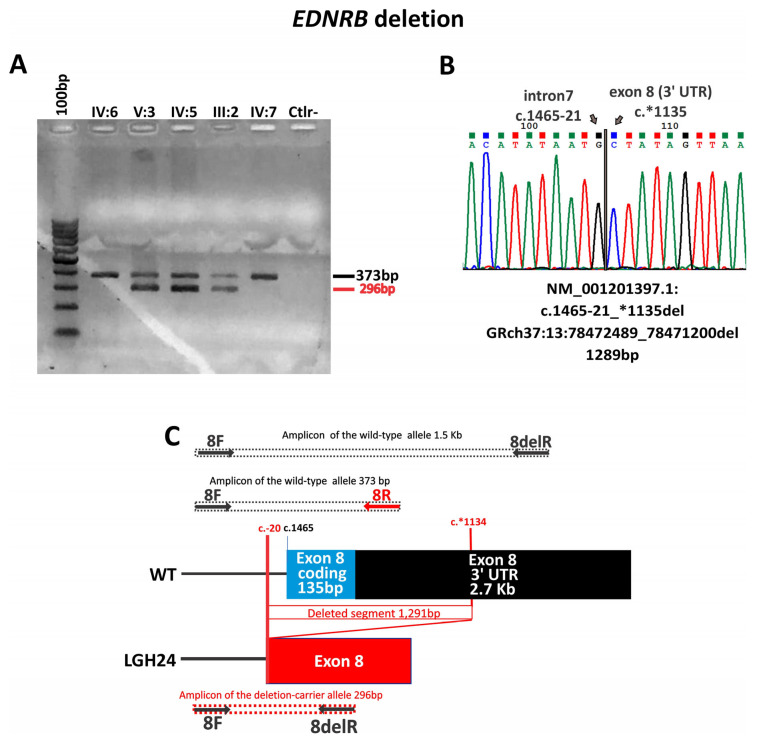
Illustration of the *EDNRB* deletion in case LGH24 (**A**) Gel electrophoresis showing the different band patterns from the *EDNRB* exon 8 deletion carriers (V:3—LGH24, IV:5, and III:2) compared to non-carriers (IV:6, IV:7); PCR conditions of the products in this gel were not suitable for the 1.5 Kb fragment. (**B**) The Sanger sequencing of the PCR product with the deletion. (**C**) Schematic representation of the deletion. Arrows indicate primers location.

As for structural variations (five cases), we found one duplication of 157 bp, three cases of exon/exons deletions, and one whole gene deletion (Table 1; Figure 2 and Figure 3). All the SNVs and indels were confirmed by Sanger sequencing. The cases with a suggestion of exon/exons (three cases) and gene deletions (one case) by WES or NGS panel after inspection of BAM files were confirmed using array-CGH (one case), MLPA (one case), and qPCR (two cases). The duplication of 157 bp in *PAX3* was detected by analyzing the soft-clipped reads on the BAM file using IGV. We found 12 misaligned reads at the exon 6 region and 11 misaligned reads on the intron that follows. Each misaligned segment had the same base pair sequence (Figure S2). The PCR for the Sanger sequencing using primers for exon 6, listed in the Methods Section, should produce an amplicon of 458 bp. The electrophoresis gel, however, showed a band of superior size when compared to control (Figure 2A). Analysis by Sanger sequencing showed a wild-type exon 6 sequence, followed by 104 base pairs of the wild-type intronic sequence, and then a repetition of the last 53 base pairs of the exon 6, followed again by the same 104 base pairs of the intronic sequence (Figure 2B,C).

The exon and gene deletions, indels, nonsense variants, and one of the splice variants are loss-of-function mutations, thus considered to be the cause of the phenotypes. The synonymous alteration (p.Thr303=) in *MITF* was predicted by SpliceAI [36] to affect splicing and it was functionally tested and demonstrated to generate a new splice site, removing the first 52 base pairs of exon 9 and generating a frameshift that adds 7 new amino acids at position 387, creating a new premature stop codon [37]. This synonymous variant can be described by its RNA alteration and final protein implication as r.859_910del and p.Glu287Valfs*8. The splice site variant detected in *MITF* (c.33+5G>A) was also analyzed by SpliceAI [36]. Although in silico predictions showed an absence of the acceptor site and a reduction in the use of the donor site in comparison with the wild type, this reduction is small and the overall impact of these sites seems to be low, deeming a final prediction of uncertain significance by SpliceAI [36] (delta score and (delta position) for AG: 0.00 (−5); AL: 0.00 (28); DG: 0.00 (32); DL: 0.15 (−5)). Also, the splice-site variant (c.484−1G>A) in *EDNRB* is a de novo variant that falls in the canonical position −1 and has an in silico score of high impact for a splice change as predicted by SpliceAI [36]. It generates a complete loss of the acceptor site (delta score and (delta position) for AG: 0.01 (−11); AL: 0.91 (−1); DG: 0.00 (49); DL: 0.03 (−37)).

**Table 1:** Causative heterozygous variants were found in 20 index cases whose first clinical diagnosis was Waardenburg Syndrome (ND = not described, NA = not applicable, ^$^ used to detect each variant).

Index Cases	Gene	Clinical Phenotype	Variant	Prediction Protein	dbSNP	Technique ^$^	ClinVar	Deafness Variation Database	Mutation Taster	ACMG	Inheritance	Segregation Analysis	Previous Description of Variants
LGH16	*MITF*—NM_000248.4	WS2	c.33+5G>A	-	-	WES	ND	ND	NA	Uncertain significance (PM2, BP4, PP1)	Familial	Segregates in the family	ND
LGH3		WS2	c.258del	p.Glu87Argfs*19	rs1576005420	WES	Likely pathogenic	ND	Disease causing	Pathogenic (PVS1, PP5, PM2)	Sporadic	Inherited from unaffected mother	ND
LGH18		WS2	c.607_608delAG	p.Arg203Alafs*10	-	WES	ND	ND	Disease causing	Likely pathogenic (PVS1, PM2, PP1)	Familial	Segregates in the family	ND
LGH26		WS2	c.610C>T	p.Gln204*	rs1559745185	WES	Likely pathogenic	Likely pathogenic	Disease causing	Pathogenic (PVS1, PP5, PP3 PM2, PP1)	Familial	Segregates in the family	ND
LGH14		WS2	Exon 5 and 6 deletion	-	-	qPCR/MLPA	ND	ND	NA	Pathogenic (PVS1)	Isolated	de novo	Same patient described in [22] as W14
LGH15		WS2	c.763C>T	p.Arg255*	rs1057517966	WES	Pathogenic/Likely pathogenic	Pathogenic	Disease causing	Pathogenic (PVS1, PM2, PP3, PP5, PP1)	Familial	Segregates in the family	[38]—Patient P44
LGH12		WS1 > WS2	c.909G>A	p.Thr303=	rs1057521096	WES	Pathogenic/Likely pathogenic	Pathogenic	Disease causing	Pathogenic (PP5, PM2, BP4, PS3)	Sporadic	NA	[37]
LGH25		WS2	Exon 8 deletion	-	-	NGS panel	ND	ND	NA	Pathogenic (PVS1)	Familial	Segregates in the family	ND
LGH5	*SOX10*—NM_006941	WS2	c.12_13delinsAT	p.Gln5*	-	WES	ND	ND	Disease causing	Pathogenic (PVS1, PM2, PP3)	Sporadic	NA	[22]—Patient W6
LGH10		WS2	c.271_275dup	p.Arg93Profs*18	-	WES	ND	ND	Disease causing	Likely pathogenic (PVS1, PM2)	Sporadic	NA	ND
LGH9	*EDNRB*—NM_000115	WS2	Whole gene deletion	-	-	WES	NA	NA	NA	Pathogenic (PVS1_Stand-alone)	Sporadic	NA	ND
LGH11		WS1 > WS2	c.484-1G>A	-	-	NGS panel	ND	ND	Disease causing	Likely pathogenic (PVS1, PM2)	Sporadic	Inherited from unaffected mother	ND
LGH17		WS2	c.898A>G	p.Met300Val	-	NGS panel	ND	ND	Disease causing	VUS (PM1, PM2)	Familial	Inherited from unaffected father	ND
LGH24		WS2	c.1465-21_*1135del Exon 8 deletion	-	-	NGS panel	ND	ND	NA	Pathogenic (PVS1, PP1)	Familial	Segregates in the family	ND
LGH22	*PAX3*—NM_181459	WS1	c.85_85+12delGGTAAGGGAGGGC	p.Val29Cysfs*81	-	WES	ND	ND	Disease causing	Likely pathogenic (PVS1, PM2)	Familial	NA	ND
LGH13		WS1	c.115A>G	p.Asn39Asp	-	WES trio	ND	ND	Disease causing	Pathogenic (PP3, PM1, PM5, PM2, PS2)	Isolated	de novo	ND
LGH21		WS1	c.896dup	p.Met299Ilefs*111	-	WES	ND	ND	Disease causing	Pathogenic (PVS1, PM2, PP1)	Familial	Segregates in the family	ND
LGH6		WS1	NC_000002.12 (NM_181459) c.958+104 (g.222221118_222221274dup)	?	-	WES	ND	ND	NA	VUS (PM2, PM4, PP4)	Sporadic	NA	ND
LGH23		WS1	c.1253del	p.Gly418Valfs*16	rs778236891	WES	ND	Unknown effect	Disease causing	Likely pathogenic (PVS1, PM2)	Familial	NA	ND
LGH1	*ACTG1*—NM_001614	WS1 > BWS2	c.277G>A	p.Glu93Lys	rs1568062529	WES trio	Likely pathogenic	Likely pathogenic	Disease causing	Pathogenic (PS2, PM1, PM2, PP2, PP3, PP5)	Isolated	de novo	ND

**Table 2:** Cohort summary including phenotypes and additional molecular analysis. + symbol indicates that the clinical feature was observed. NA = not available.

Clinical Features									Additional Molecular Analysis				
Family ID	Dystopia Canthorum	Eye Pigmentation Abnormality	Hair Pigmentation Abnormality	Skin Pigmentation Abnormality	Hearing Impairment	WS Type	Proband	Mutation Segregation	NGS Panel	Exome Trio	MLPA (*PAX3, MITF, SOX10*)	Array-CGH	Comments
LGH1	+	Blue eyes	−	−	+	1 > BWS2	Sporadic	de novo	−	+	+	−	
LGH2	−	Bright blue iridis	−	−	+	2	Sporadic	Unsolved case	+	+	+	+	
LGH3	−	Bright blue iridis	+	−	+	2	Sporadic	Inherited unaffected mother	−	−	+	−	
LGH4	−	Heterochromia iridis	+	+	+	2	Sporadic	Unsolved case	+	+	+	−	
LGH5	−	Heterochromia iridis	+	−	+	2	Sporadic	NA	−	−	+	−	
LGH6	+	Bilateral heterochromia iridis	+	−	+	1	Sporadic	NA	−	−	+	+	Ala nasi hipoplasia
LGH7	−	Bright blue iridis with brown spotting	+	−	+	2	Sporadic	Unsolved case	+	+	+	+	
LGH8	−	−	+	−	+	2	Sporadic	Unsolved case	−	−	+	−	Moderate mixed (R) and conductive (L) HL and not included for the NGS panel
LGH9	−	Heterochromia iridis and bright blue iridis	+	+	+	2	Sporadic	NA	−	−	+	+	Deletion suspicion by WES and confirmed with array-CGH
LGH10	−	Bright blue iridis	−	−	+	2	Sporadic	NA	−	−	+	−	
LGH11	Apparent	Heterochromia iridis and bright blue iridis	−	−	+	1 > 2	Sporadic	Inherited from unaffected mother	+	+	−	+	
LGH12	Apparent	Heterochromia iridis and bright blue iridis	−	−	+	1 > 2	Sporadic	NA	−	−	−	−	
LGH13	+	Heterochromia iridis	+	−	+	1	Sporadic	de novo	−	+	−	−	Ala nasi hipoplasia
LGH14	−	Heterochromia iridis	−	+	+	2	Sporadic	de novo	−	+	+	−	Nasal root hyperplasia. Normal MRI, CT-scan. Patient W14 [22]
LGH15	−	Bright blue iridis	−	−	+	2	Familial	+	−	−	+	−	
LGH16	−	Heterochromia iridis	−	−	+	2	Familial	+	−	−	+	+	
LGH17	−	Heterochromia iridis	−	−	+	2	Familial	Inherited from unaffected father	+	−	+	+	
LGH18	−	Bright blue iridis	+	−	+	2	Familial	+	−	−	+	−	
LGH19	−	−	+	+	+	2	Familial	Unsolved case	+	−	+	+	
LGH20	−	Bright blue iridis	+	−	+	2	Familial	Unsolved case	+	−	+	+	
LGH21	+	Bright blue iridis	+	−	+	1	Familial	+	−	−	−	−	Ala nasi hipoplasia
LGH22	+	Heterochromia iridis and bright blue iridis	+	−	+	1	Familial	NA	−	−	+	−	Ala nasi hipoplasia
LGH23	+	Heterochromia iridis and bright blue iridis	+		+	1	Familial	NA	−	−	+	−	Ala nasi hipoplasia
LGH24	−	Heterochromia iridis and bright blue iridis	−	−	+	2	Familial	+	+	−	−	+	Normal MRI and CT. Patient W12 [22]
LGH25	−	Bright blue iridis	−	−	+	2	Familial	+	+	−	−	+	Normal MRI and CT. Patient W13 [22]
LGH26	−	Heterochromia iridis and bright blue iridis	−	−	+	2	Familial	+	−	−	+	−	

## 4. Discussion

The causes for WS types 1 and 3 are restricted to the *PAX3* gene, while WS4 cases are around seventy percent explained by pathogenic mutations on *SOX10*, *EDNRB*, and *EDN3* [10]. As for WS2, clinical and molecular diagnoses are more challenging. The reason is that type 2 is very genetically heterogeneous and lacks a remarkable, highly penetrant, distinctive phenotype feature (i.e., dystopia canthorum (DC) in WS1; DC and limb defects in WS3 and Hirschsprung disease in WS4). Most pathogenic mutations are found in the *MITF* and *SOX10* genes. In fewer cases, the cause for WS2 can also be found in *EDNRB* [10,13]. Moreover, screening *EDN3* for WS2 might resolve some cases, since heterozygous relatives of WS4 patients with variants on either *EDNRB* or *EDN3* can present some symptoms of WS2 patients, and because of the close functional relationship between the products of both genes [16].

After the sole case correlating the *KITLG* gene to WS2 in the heterozygous state [14], Vona et al. (2022) recently confirmed the role of the gene in WS2 in additional four families carrying three different homozygous variants. Conversely, more than two decades after the publication of two unrelated patients carrying homozygous deletions on *SNAI2* as the cause of WS2 [39], researchers retracted their conclusion, citing the limitations of the technologies (Southern blotting and RT-qPCR) used in the study [40]. They now believe that their results were an artifact of these technologies. Also, another patient in ClinVar with a heterozygous deletion that includes the whole *SNAI2* gene did not present a WS-like phenotype, allowing the conclusion that it does not cause WS in heterozygosis either. Altogether, *SOX10*, *MITF*, *EDNRB*/*EDN3*, and now *KITLG*, account for approximately only half of the clinically diagnosed WS2 patients [10,13].

Among our 26 probands, eight and 18 were initially diagnosed as WS1 and WS2, respectively. Of the eight suspected as WS1, five were confirmed by molecular diagnosis (62%) and associated with the *PAX3*. However, three were reassigned: two WS2 patients and one BWS2 patient. These findings emphasize the value of molecular diagnosis to accurate diagnosis and, therefore, management of additional symptoms and genetic counseling.

For WS2, 12 out of 18 (66%) patients had their molecular cause detected and the initial clinical suspicion confirmed. Six cases remained unsolved.

WES solved 15 cases (58%). The targeted NGS panel solved four additional cases (44%). The non-detection of these four cases by WES can be explained by lower coverage and depth when compared to the panel.

The higher detection rate for WS1 patients is in concordance with a well-established clinical diagnosis, a highly penetrant phenotypical feature (i.e., DC), and a restricted genetic cause [10]. The inaccurate diagnosis of WS1 in those two WS2 patients is due to lack of measurements of their W-index, and an apparent DC was noted. Reevaluation of these probands later in life to confirm the persistence of the DC would have most certainly proved untrue, hence allowing the correction of the clinical diagnosis. Similarly, some reports have mistakenly highlighted the involvement of pathogenic variants in *EDNRB* and *SOX10* as causes of WS1, the former in the heterozygous [26] and homozygous state [41], and the latter in the heterozygous state [42]. These authors made use of the Caucasian-based W-index, proposed by Arias and Mota in 1978 [43], to establish the clinical difference between types 1 and 2 among Asian patients. This index takes into account facial ocular measurements to define the presence of DC. An adjustment of the threshold of the W-index is needed for the Asiatic population [44,45]. However, in a Western population, the W-index showed 60% and 93% discrimination between WS1 and WS2 cases, respectively [6].

The WS1 misdiagnosed case now reassessed as BWS2 was solved by the WES analysis of the trio. With few cases described in the literature, BWS was first described in 1988 [46], characterized by the combination of iris coloboma, bilateral ptosis, hypertelorism, wide nasal bridge, and prominent epicanthus. Sensorineural deafness and altered measurements of the orbital region are common to both BWS and WS [47]. The previous diagnostic hypothesis of WS1 was based on the occurrence of deafness associated with the impression of DC, with a W index of 2.02, a value contained within the diagnostic indecision zone between WS1 and WS2 that ranges from 1.95 to 2.07 [2]. In addition, the blue irises exhibited brown spots that surrounded the pupillary border, giving an impression of bilateral heterochromia iridis, which was refuted after appropriate ophthalmological evaluation.

### 4.1. PAX3 Variants

In this study, among the five variants found in the *PAX3* gene, three of them are loss of function (LoF) variants (p.Met299Ilefs*111, p.Val29Cysfs*81, p.Gly418Valfs*16) and therefore were considered as the molecular cause of the WS1 clinical diagnosis (Table 1). The sole missense variant (p.Asn39Asp) has never been published in populational databases, but a change in the same amino acid position for a tyrosine has already been considered causative and associated with the WS1 phenotype in a familial case [10]. The intragenic duplication in *PAX3* (NC_000002.12 (NM_181459) c.958+104 (g.222221118_222221274dup)) was an interesting finding. However, since it is an intronic duplication, one cannot be sure whether it would be removed by splicing, without functional consequences. Nevertheless, analysis of the duplicated allele in a splice site prediction tool (BDGP—https://www.fruitfly.org/—accessed on 7 December 2023) predicted that two novel splice sites (one acceptor and one donor) would be potentially formed by the duplicated segment. These novel splice sites could lead to aberrant transcripts with intron and duplication retention after splicing. This favors the hypothesis that the duplication is causative, and this could be clarified by RNA studies.

### 4.2. MITF Variants

The eight variants found in the *MITF* gene include six LoF (p.Glu87Argfs*19, p.Arg255*, p.Arg203Alafs*10, p.Gln204*, exon 5–6 deletion, exon 8 deletion) and can be considered as the molecular cause of WS2 in those patients. However, two of the LoF variants were found in asymptomatic relatives: one asymptomatic out of three relatives that carried the p.Glu87Argfs*19 variant (LGH3), and one asymptomatic out of eight individuals that carried the p.Arg255* variant (LGH 15) (Supplementary Figure S1).

Cases of incomplete penetrance and variable expressivity were documented for *MITF* [10,48]. Regarding the other two variants, the synonymous variant was also considered to be causative of the phenotype since it had previously been described and a functional assay revealed the generation of a new splice site, that originates a new premature stop codon [37]. The last *MITF* variant is a splice-site change (c.33+5G>A) that segregates within the family in two other individuals (one asymptomatic and the other presenting hearing impairment and iris depigmentation). The variant is absent in seven asymptomatic family members, which strongly argues in favor of this variant being the cause of the WS2 phenotype (Figure S1). Furthermore, a variant at the same position but changing from G>C is reported on ClinVar, with three submitted interpretations as pathogenic in WS2 in both heterozygous [49,50] and homozygous states [51]. Despite in silico predictions indicating a reduction in the use of a donor site, the final prediction was of uncertain significance by SpliceAI [36].

### 4.3. SOX10 Variants

In our cohort, only two patients had variants in *SOX10*, both being LoF (c.12_13delinsAT; p.Gln5* and c.258del; p.Arg93Profs*18). Initiation of translation could occur in another in-frame initiation codon, such as Met90, in the first case. However, in a functional analysis of an in-frame *SOX10* protein produced with a translation initiation using Met90, the first in-frame methionine of *SOX10*, it was demonstrated that the protein was not functional [52]. Even if in the first case of LoF variant (c.12_13delinsAT; p.Gln5*) it was possible to translate the protein from Met90, it can be speculated that it would be nonfunctional. This putative non-functional protein conserves the HMG and transactivation domains but lacks most of the dimerization domain. The specific clinical signs of this patient are consistent with WS2: profound unilateral deafness, white frontal forelock, W index = 1.58, and heterochromia iridis. Curiously the same variant (c.12_13delinsAT) was also described in another Brazilian patient with WS2 [22], who inherited it from his affected mother. The present case is sporadic, but a de novo occurrence was not confirmed. The proband carrying the c.12_13delinsAT, reported by Batissoco et al. (2022) [22], has blue hypoplastic iridis, profound sensorineural hearing loss, and imaging exams revealed vestibular and semicircular canals dysplasia, while his mother has unilateral hearing loss associated with hair and eye pigmentation abnormalities, as seen in the present patient with the same variant. The hypothesis of relatedness between these two cases could not be ruled out.

### 4.4. EDNRB Variants

Lastly, four variants were found in the *EDNRB* gene, three of them being LoF (whole gene deletion, exon 8 deletion, canonical splice-site variant).

In the familial case with the exon 8 deletion (c.1465-21*1135del) (Figure 3), there are at least three documented non-penetrant patients. The proband’s father has both eyes with different shades of brown and the proband’s brother was born with a white forelock that disappeared with time (both were considered as affected). Cases of incomplete penetrance and variable expressivity involving the *EDNRB* gene are long known [10,19,53,54,55].

The splice-site variant (c.484−1G>A) is a de novo variant that was predicted to generate a complete loss of the acceptor site, thus is considered as causative.

The fourth variant, a missense (p.Met300Val), was detected in a familial case. The sister of the proband presents heterochromia iridis, while the carrier father is asymptomatic. The amino acid at position 300 and the adenine base that initiates this methionine codon are highly conserved among different species. Nonetheless, only functional analysis could confirm the impact of this variant. These could be performed by immunofluorescence in transfected cells, comparing wild-type and mutant subcellular localization, calcium mobilization assay, and G protein activation and correct transmission of the signal to downstream pathways [13,56,57,58].

### 4.5. Final Considerations

The overall rate of detection of the causative variants was 77%, with 8 out of 26 cases (30%) presenting with novel variants, never described in the databases or the published literature. It is very difficult to compare the CNVs to other studies, given the difficulty of characterizing precisely the breakpoints, and to compare them with the literature.

In our study, NGS contributed to correcting the initial suspected clinical diagnostic in three probands (LGH11, LGH12, and LGH1) that had a previous diagnosis of WS1. For the first two, the targeted panel and WES revealed variants in *EDNRB* and *MITF*, respectively, indicating they were WS2 cases. The latter proband had a de novo variant in *ACTG1* and retrospective phenotyping allowed us to confirm the clinical diagnosis as BWS2.

NGS, either in the form of WES or panels, clearly allowed changing the order and choice of genetic tests. Here we showed that we were able to detect CNVs without MLPA. The only six samples not studied by MLPA were LGH11, LGH12, LGH13, LGH21, LGH24, and LGH25. As can be observed in Table 1, all these cases were solved with NGS. The lack of MLPA in these cases did not affect the overall rate of detection of causative variants reported in our study.

Finally, six patients (23%), all WS2, remained without identification of the causative variant, a frequent finding in other cohorts [13,22]. The efforts in trying to find the molecular diagnosis should focus on broad genetic tests that can verify a large number of genes and non-coding regions, while also maintaining good quality for reliable analysis. NGS panels cover many genes and usually maintain a better depth of coverage when compared to WES or WGS. Heterogeneous coverage explains why we, unfortunately, missed some variants in exomes but were able to detect them after the NGS panel. On the other hand, WGS can cover more regions, which allows for the discovery of novel genes or novel mutational mechanisms, including non-coding regions. The fraction of cases without molecular explanation is indicative of the possibility of alterations in more than one gene (oligogenic inheritance) or that other unknown mechanisms or genes may play a role in those unsolved cases.

## Data Availability

The data that supports the findings of this study are available on request from the corresponding author. The data are not publicly available due to privacy or ethical restrictions.

## Supplementary Materials

The following supporting information can be downloaded at: https://www.mdpi.com/article/10.3390/audiolres14010002/s1, Figure S1: Pedigrees of the cases with the clinical characterization and the segregation of the variants; Figure S2: Visualization in IGV of the misaligned soft-clipped reads of proband LGH6.
