# Supplementary material for: Waardenburg Syndrome: The Contribution of Next-Generation Sequencing to the Identification of Novel Causative Variants
Source: Audiol Res. 2023 Dec 21;14(1):9–25. doi: 10.3390/audiolres14010002 (PMC10886116; doi:10.3390/audiolres14010002)
Supplement: Supplementary file 1 [file audiolres-14-00002-s001.zip › audiolres-2668071-supplementary.pdf]

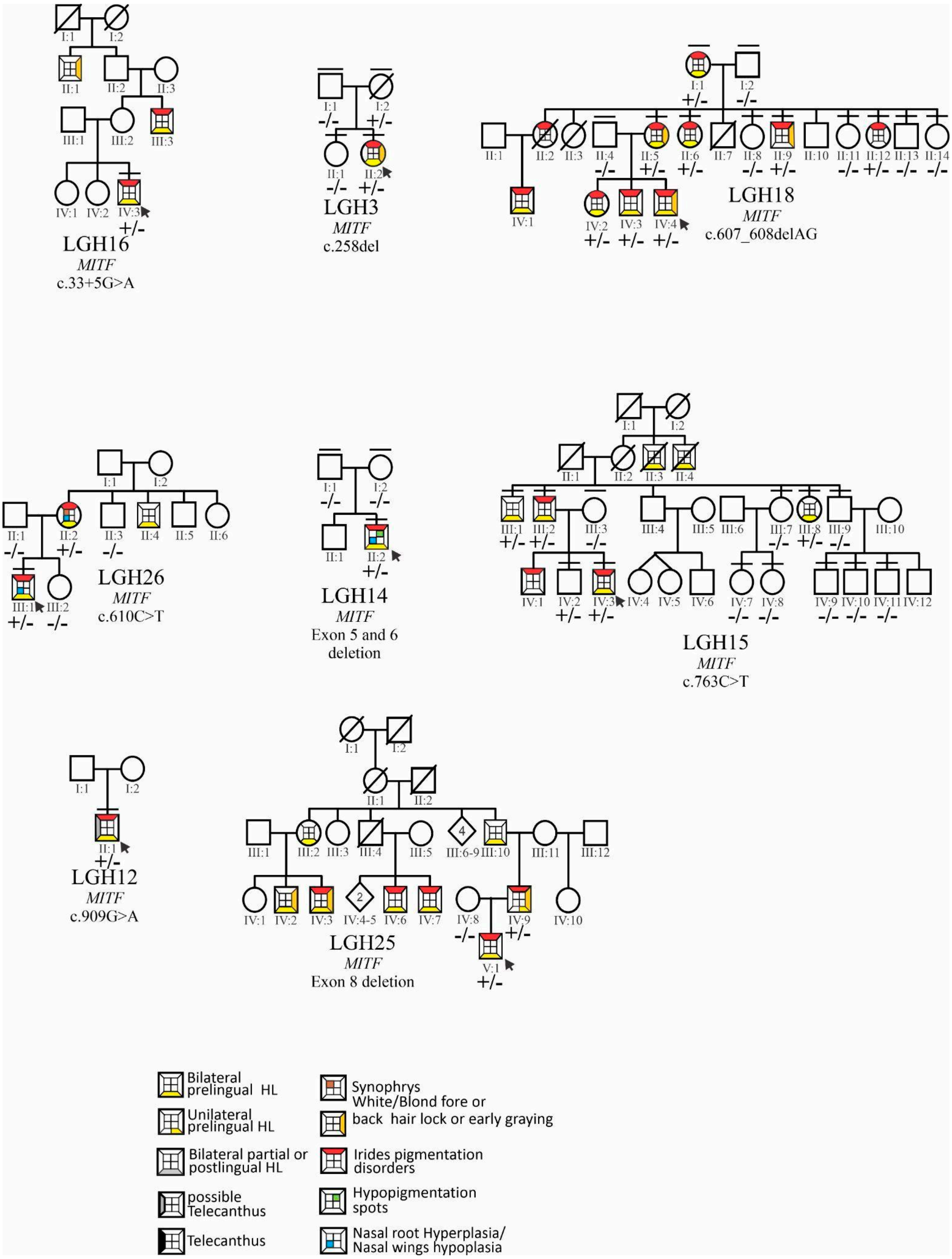

Figure S1: Pedigrees of the cases with the clinical characterization and the segregation of the variants

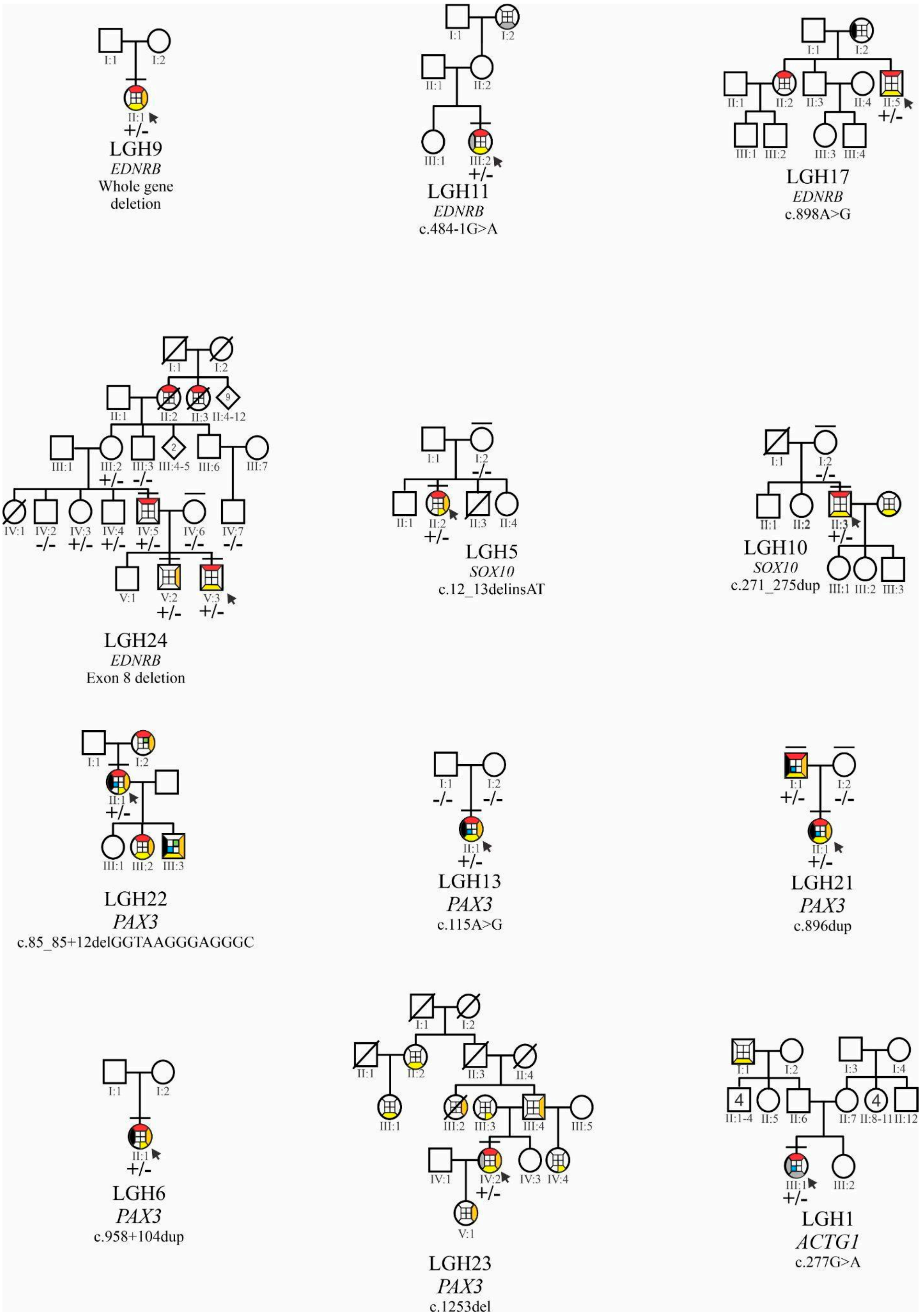

Continuation of Figure S1

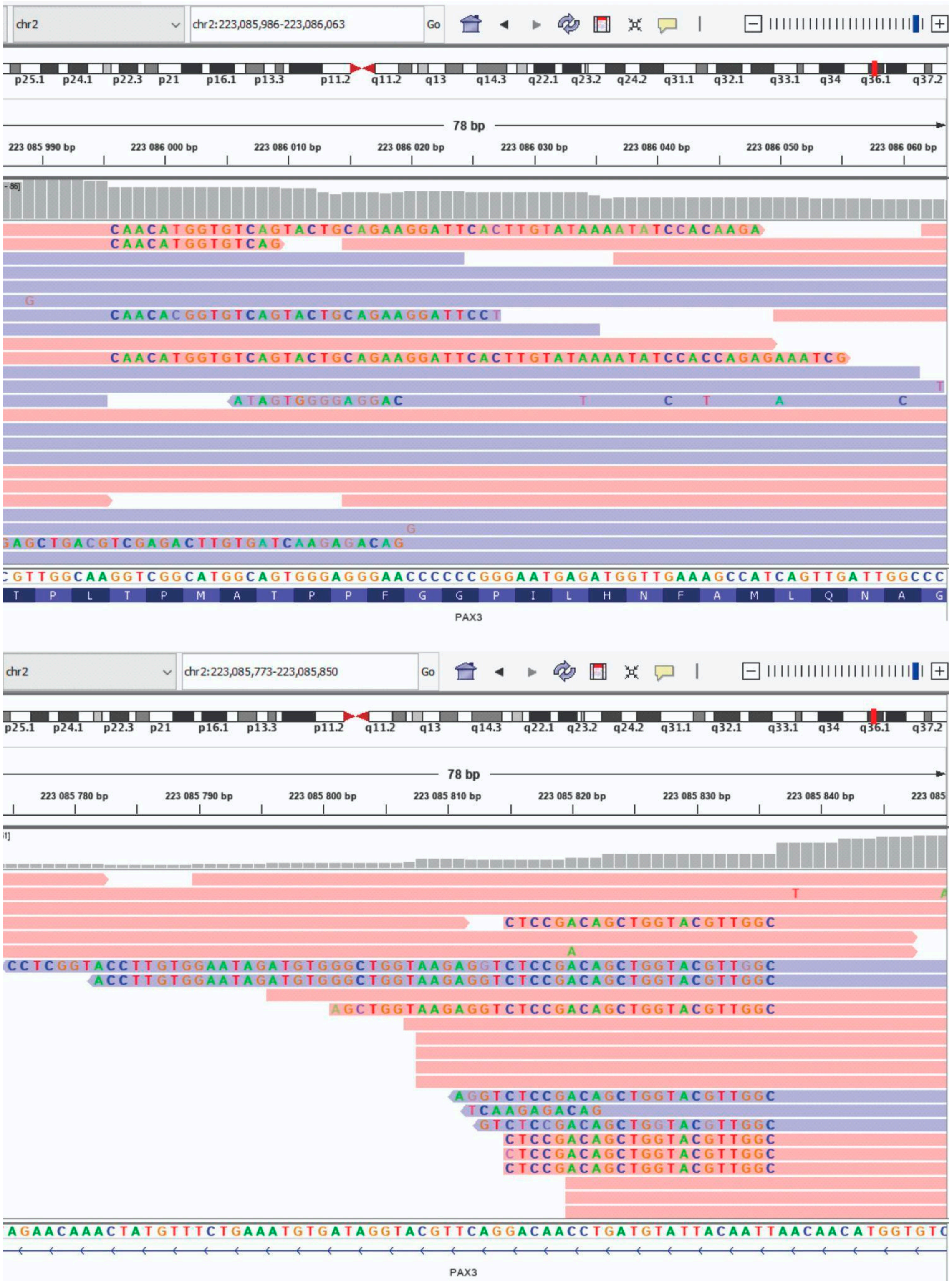

Figure S2: Visualization in IGV of the misaligned soft-clipped reads of proband LGH6.
